# Platelet to lymphocyte ratio as a predictive factor of 30-day mortality in patients with acute mesenteric ischemia

**DOI:** 10.1371/journal.pone.0219763

**Published:** 2019-07-17

**Authors:** Emmanuel Augène, Fabien Lareyre, Julien Chikande, Lucas Guidi, Ali Ballaith, Jean-Nicolas Bossert, Yann Pelletier, Caroline Caradu, Réda Hassen-Khodja, Juliette Raffort

**Affiliations:** 1 Department of Vascular Surgery, University Hospital of Nice, Nice, France; 2 Université Côte d’Azur, CHU, Nice, France; 3 Unit of Vascular Surgery, CHU de Bordeaux, University of Bordeaux, Bordeaux, France; 4 Clinical Chemistry Laboratory, University Hospital of Nice, Nice, France; Massachusetts General Hospital, UNITED STATES

## Abstract

**Introduction:**

Acute mesenteric ischemia is associated with high rates of mortality. The aim of this study was to investigate the prognostic value of the neutrophil to lymphocyte ratio (NLR) and the platelet to lymphocyte ratio (PLR) on 30-day outcomes in patients with acute mesenteric ischemia.

**Material and methods:**

Consecutive patients who were admitted for an acute mesenteric ischemia were retrospectively included. The full white blood count at the time of admission to the hospital was recorded. The population was divided into 4 subgroups according to the quartiles of the NLR and the PLR. The 30-day outcomes including the mortality and the complications were compared among the subgroups.

**Results:**

In total, 106 patients were included. A surgical treatment including revascularization and/or digestive resection was performed for 56 patients (52.8%). The 30-day all-cause mortality was 72 patients (67.9%). Patients with higher PLR value (PLR >429.3) had significantly higher rate of mortality compared to the other groups (80.8% vs 46.2%, 66.7% and 77.8%, p = 0.03). No significant difference on 30-day outcome was observed among the subgroups divided according to the NLR.

**Conclusion:**

The PLR, but not the NLR, is a predictive factor of 30-day mortality in patients with acute mesenteric ischemia.

## Introduction

Acute mesenteric ischemia is characterized by a significant decrease in mesenteric blood flow which can cause irreversible changes in the intestinal mucosa and lead to intestinal necrosis, peritonitis, severe sepsis and multiple organ failure [1, 2]. Early diagnosis of the disease is essential as the duration of the ischemia appears as a major prognostic factor [1]. Although advances in the diagnosis and the treatment have been achieved over the past few years, the disease is still associated with high rates of mortality, ranging from 40 to 70% [3]. The identification of diagnostic and prognostic factors may help to improve care provided to patients. Mesenteric ischemia is associated with an acute inflammatory response and several biomarkers have demonstrated their interest in the diagnosis of the disease including serum lactic acid deshydrogenase, D-dimer, intestinal fatty acid-binding protein (I-FABP), α-glutathione S-transferase (α-GST), D-lactate or ischemia modified albumin (IMA) [4–7].

The neutrophil to lymphocyte ratio (NLR) has been identified as an inflammatory biomarker and its interest has been demonstrated in a wide range of pathologic states including cardiovascular and ischemic diseases [8–13]. The platelet to lymphocyte ratio (PLR) is another indicator of the systemic inflammatory response and several studies have pointed its interest as a biomarker in cardiovascular diseases including coronary artery disease, peripheral arterial occlusive disease or abdominal aortic aneurysm [14–16]. The interest of the NLR or the PLR in the diagnosis of acute mesenteric ischemia has been demonstrated by several studies [17–19]. However, their prognostic value has been so far scarcely investigated. The aim of this study was to investigate the predictive value of the NLR and the PLR on the outcomes of patients diagnosed with an acute mesenteric ischemia.

## Material and methods

### Population

Consecutive patients who were admitted for an acute mesenteric ischemia were retrospectively included at the University Hospital of Nice between January 2011 and February 2019. The study was conducted according to the World Medical Association Declaration of Helsinki and was approved by the University Hospital of Nice review board. Written informed consent was waived by the review board because of the retrospective nature of the study. All subjects were anonymized when included in the study.

Inclusion criteria were patients older than 18 years-old diagnosed for an acute mesenteric ischemia at the Emergency Department. Patients who had a history of chronic mesenteric ischemia, infectious colitis or known hematologic disorders were excluded. Acute mesenteric ischemia was defined as a sudden decrease in blood supply to the bowel, according to the guidelines of the World Society of Emergency Surgery [20]. The diagnosis was made by the radiologist and the visceral surgeon based on the association of clinical symptoms, biological disorders and radiologic signs on CT-Scan. Clinical symptoms included the presence of intestinal angina, abdominal pain, vomiting, peritonitis or abdominal guarding, severe sepsis or septic shock, rectorrhagia or melena. Patients general characteristics were recorded including the age, the body mass index, the presence of cardiovascular risk factors or chronic diseases. The localization of the thrombus was assessed on injected CT-scan images by two independent observers. The full white blood count at the time of admission to the hospital was recorded.

The full white blood count at the time of admission to the Emergency Department was recorded. Every patient suspect for an acute mesenteric ischemia had a biological investigation including a full white blood count at the in-hospital admission. We retrospectively collected the results from the first blood test harvested at the admission to the Emergency Department. The neutrophil to lymphocyte ratio (NLR) was calculated by dividing the absolute value of neutrophils to lymphocytes; the platelet to lymphocyte ratio by dividing the absolute value of platelets to lymphocytes (PLR). The glomerular filtration rate was calculated according to the Chronic Kidney Disease EPIdemiology collaboration (CKD-EPI) equation. The treatment for the acute mesenteric ischemia was collected including the technic for revascularization (thrombectomy, angioplasty or bypass) and the type of digestive resection when necessary (jejunal, ileal or colic resection). Therapeutic abstention was defined as the absence of surgical intervention to treat the acute mesenteric ischemia.

### Data collection

Clinical data and procedural characteristics were collected using electronic and manuscript medical records. The electronic record was collected using a software named Clinicom. Patients were retrospectively searched in the software using the diagnostic code K55.0, defined as “acute vascular disorders of the intestine” by International Classification of Diseases (ICD 10) of the World Health Organization (WHO). They were then selected according to the inclusion criteria. Imaging data were recorded and extracted from the soft-wares Picture Archiving and Communication Systems (PACS) and Aquarius iNtuition Edition version 4.4.8 (TeraRecon,Inc. San Mateo).

### Study end points

To evaluate the potential interest of the NLR and the PLR as predictive factors of patients diagnosed with an acute mesenteric ischemia, the population was divided into quartiles according to the values of the NLR and the PLR. The clinical and procedural characteristics as well as the 30-day outcomes including the mortality and the complications were compared among the subgroups. Complications were categorized accordingly to the affected organ. Vascular complications were defined as a failure of revascularization and/or an extension of the intestinal necrosis.

### Statistical analysis

Categorical data were expressed as the number of patients and percentage and continuous variables were expressed as the median with interquartile range. The Chi-square test was used to compare group differences for categorical data. A one-way analysis of variance (ANOVA) was used for continuous variables. A two-sided P value <0.05 was considered as significant. Statistical analyses were performed using GraphPad Prism software (version 7.00, La Jolla California USA). A post hoc analysis to compute the achieved power of the study was performed using z-tests with power (1 - β) set at 0.80 and α = 05, two-tailed, using G*Power software (version 3.1.9.4, Faul & Erdfelder).

## Results

During the inclusion period, 106 patients were diagnosed with an acute mesenteric ischemia (Table 1). The median of the age was 79 years (interquartile range: 61–88) and 47 patients (44.3%) were men. At the in-hospital admission, the leukocyte count was 15.8 * 10^9/L (11.6–21.9), the neutrophils 13.2 * 10^9/L (9.6–19), the lymphocytes 1 * 10^9/L (0.8–1.6). The median NLR was 14.2 (7.5–21.2) and the PLR was 268.1 (167.9–429.3). The acute mesenteric ischemia was caused by a thrombosis of the mesenteric superior artery for 77 patients (72.6%), of the mesenteric inferior artery for 13 patients (12.3%), the celiac trunk in 10 patients (9.4%) or the mesenteric vein for 4 patients (3.8%). A surgical treatment was performed for 56 patients (52.8%). Intestinal resection of the jejunum was required for 7 patients (6.6%), of the ileum for 26 patients (24.5%) and of the colon in 27 cases (25.5%). A revascularization was performed using a thrombectomy for 12 patients 11.3%), an endovascular repair for 5 patients (4.7%) or a bypass for 6 patients (5.7%). The all-cause 30-day mortality was 72 patients (67.9%).

**Table 1:** Characteristics of patients diagnosed with an acute mesenteric ischemia.

Patients characteristics (n = 106)		Data
General characteristics	Age	79 (61–88)
	Male sex	47 (44.3%)
	Body mass index (kg/m^2^)	22.1 (20–25.9)
	Diabetes	22 (20.8%)
	Arterial hypertension	63 (59.4%)
	Dyslipidemia	9 (8.5%)
	Smoking	31 (29.2%)
Symptoms	Intestinal angina	169 (75.4%)
	Pain	29 (12.9%)
	Vomiting	25 (11.2%)
	Peritonitis	57.4 +/ 13.8
	Shock	113 (50.4%)
	Rectal hemorrhage	111 (49.6%)
Localization of the thrombosis	Celiac trunk	10 (9.4%)
	Mesenteric superior artery	77 (72.6%)
	Mesenteric inferior artery	13 (12.3%)
	Mesenteric vein	4 (3.8%)
	Undetermined	13 (12.3%)
Biological parameters at in-hospital admission	Red blood cells (* 10^12/L)	4.5 (3.9–5)
	Hemoglobin (g/dL)	13 (10–14.5)
	Thrombocytes (*10^9/L)	268.5 (199.3–373)
	Leukocytes (*10^9/L)	15.8 (11.6–21.9)
	Neutrophils (*10^9/L)	13.2 (9.6–19)
	Lymphocytes (*10^9/L)	1 (0.8–1.6)
	NLR	14.2 (7.5–21.2)
	PLR	268.1 (167.9–429.3)
	eGFR (mL/mn/1.73m^2^)	26 (22.5–64.5)
Surgical treatment	Intestinal resection	60 (56.6%)
	- Jejunum resection	7 (6.6%)
	- Ileum resection	26 (24.5%)
	- Colon resection	27 (25.5%)
	Revascularization	23 (27.7%)
	- Vascular bypass	6 (5.7%)
	- Endovascular repair	5 (4.7%)
	- Thrombectomy	12 (11.3%)
	Surgical therapeutic abstention	50 (47.2%)

Values are median (interquartile range) or n (%).

eGFR: estimated glomerular filtration rate

NLR: neutrophil to lymphocyte ratio

PLR: platelet to lymphocyte ratio

EVAR: endovascular aortic repair

To evaluate the potential interest of the PLR in patients diagnosed with an acute mesenteric ischemia, the population was divided into 4 subgroups according to the quartile of the PLR: group I (PLR<167.9), group II (167.9<PLR<268.1), group III (268.1<PLR<429.3) and group IV (PLR>429.3). The localization of the thrombosis did not differ among the groups and was in the superior mesenteric artery in respectively 18 patients (69.2%), 21 (77.8%), 17 (63%) and 21 (80.8%), p = 0.45 (S1 Table). The type of surgical treatment including the technique for revascularization or the need of intestinal resection did not significantly vary among the groups. However, in group IV (PLR>429.3), the proportion of surgical therapeutic abstention was significantly higher compared to the other groups (57.7% vs 23.1%, 51.9% and 55.6%, p = 0.04). The outcomes of patients were compared according to the PLR value at in-hospital admission (Table 2). The 30-day mortality rate was significantly higher in group IV (PLR>429.3) compared to the other groups (80.8% vs 46.2%, 66.7% and 77.8%, P = 0.03).

**Table 2:** Outcomes of patients with digestive ischemia according to the PLR value.

Outcomes	PLR<167.9 (n = 26)	167.9<PLR<268.1 (n = 27)	268.1<PLR<429.3 (n = 27)	PLR>429.3 (n = 26)	P value
30-day mortality	12 (46.2%)	18 (66.7%)	21 (77.8%)	21 (80.8%)	0.03
Hemorrhagic complications	1 (3.8%)	1 (3.7%)	0 (0%)	1 (3.8%)	0.82
Cardiac complications	2 (7.7%)	4 (14.8%)	2 (7.4%)	1 (3.8%)	0.54
Vascular complications	5 (19.2%)	1 (3.7%)	3 (11.1%)	2 (7.7%)	0.26
Neurologic complications	2 (7.7%)	1 (3.7%)	2 (7.4%)	2 (7.7%)	0.92
Respiratory complications	4 (15.4%)	3 (11.1%)	3 (11.1%)	3 (11.5%)	0.96
Renal complications	4 (15.4%)	1 (3.7%)	0 (0%)	1 (3.8%)	0.08
Infections	4 (15.4%)	3 (11.1%)	5 (18.5%)	5 (19.2%)	0.85

Values are expressed as n (%).

PLR: platelet to lymphocyte ratio

The potential interest of the NLR as a predictive factor was then evaluated and patients were divided into 4 other subgroups according to the NLR value: group I (NLR<7.5), group II (7.5<NLR<14.2), group III (14.2<NLR<21.2) and group IV (NLR>21.2) (S2 Table). No significant difference was observed among the subgroups regarding the localization of the thrombosis, the type of surgical treatment or the rate of surgical therapeutic abstention. The outcome of patients did not significantly differ among the subgroups (Table 3). Even if patients from group IV tended to have higher 30-day mortality rate compared to the other groups, the difference did not reach statistical significance (76.9% vs 53.8%, 70.4% and 70.4%, p = 0.57).

**Table 3:** Outcomes of patients with acute mesenteric ischemia according to the NLR value.

Outcomes	NLR<7.5 (n = 26)	7.5<NLR<14.2 (n = 27)	14.2<NLR<21.2 (n = 27)	NLR>21.2 (n = 26)	P value
30-day mortality	14 (53.8%)	19 (70.4%)	19 (70.4%)	20 (76.9%)	0.57
Hemorrhagic complications	0 (0%)	1 (3.7%)	1 (3.7%)	1 (3.8%)	0.80
Cardiac complications	2 (7.7%)	3 (11.1%)	3 (11.1%)	1 (3.8%)	0.75
Vascular complications	4 (15.4%)	4 (14.8%)	1 (3.7%)	2 (7.7%)	0.43
Neurologic complications	1 (3.8%)	2 (7.4%)	2 (7.4%)	2 (7.7%)	0.94
Respiratory complications	4 (15.4%)	5 (18.5%)	3 (11.1%)	1 (3.8%)	0.39
Renal complications	3 (11.5%)	0 (0%)	2 (7.4%)	1 (3.8%)	0.31
Infections	4 (15.4%)	6 (22.2%)	2 (7.4%)	5 (19.2%)	0.48

Values are expressed as n (%).

NLR: neutrophil to lymphocyte ratio

To investigate the potential impact of the initial characteristics of the patients on the results, the prevalence of comorbidities was compared among the 4 subgroups classified according to the PLR and the NLR values (S3 and S4 Tables). No significant difference was found regarding the presence of diabetes, arterial hypertension, smoking, history of inflammatory diseases or history of cancer. However, patients with PLR>429.3 were significantly older than the other subgroups (84 vs 67, 78 and 83 years-old, P = 0.03).

At last, a post hoc analysis was performed to compute the achieved power of the study. For the PLR, the statistical power of the study to detect a difference between group I (PLR<167.9) and group IV (PLR>429.3) was 76%. For the NLR, the statistical power to detect a difference between group I (NLR<7.5) and group IV (NLR>21.2) was 41%. Even though patients from group IV tended to have a higher rate of mortality, the results did not reach statistical significance. Power analysis revealed that a sample of 66 patients per group would be required to detect an effect for the NLR.

## Discussion

Early diagnosis and treatment of acute mesenteric ischemia are critical to limit irreversible changes to the intestinal mucosa [21]. A better identification of patients with high risk of complications may help to choose the most appropriate therapeutic approach. In this study, the PLR at in-hospital admission, but not the NLR, was predictive of the 30-day mortality.

A high platelet count is often associated with inflammatory process as inflammatory mediators stimulate megakaryocytic proliferation [19]. In contrast, a decrease of lymphocytes occurs, mainly induced by steroid exposure [22]. As a consequence, the PLR has been proposed as an inflammatory marker and several authors have shown a positive correlation between the PLR and C-reactive protein (CRP) concentration [14, 19]. Several studies have demonstrated the association of the PLR with patients’ outcomes during other ischemic diseases. The PLR was independently and positively associated with the severity of coronary atherosclerosis (r = .370, P < .001) [14]. In addition, high-PLR values were associated with worse outcomes, increased rates of insufficient recanalization and higher size of infarcted area in patients with acute ischemic stroke [23]. The interest of the PLR in acute mesenteric ischemia has been so far poorly investigated. A first study revealed that patients with acute mesenteric ischemia had significantly higher PLR levels than healthy control individuals [19]. A PLR value higher than 157 yielded an area under the curve of 0.604 (95% confidence interval 0.486–0.722, sensitivity 59%, specificity 65%), suggesting the potential interest of the marker in the diagnosis of acute mesenteric ischemia. Other studies pointed its interest as a prognostic factor. A study involving 137 patients with acute mesenteric arterial embolism or thrombosis compared those who had a poor outcome (cases of intestinal necrosis or death) and those with a better outcome (cases without intestinal necrosis who survived) [24]. The PLR was identified as an independent prognostic factor (OR = 4.871, 95% confidence interval: 1.627–14.587, P = 0.005). Another study involving 34 patients with acute mesenteric ischemia revealed that the PLR was significantly higher in patients who died compared to those who survived (373.8 vs 288.5, P = 0.045) [25]. Even if the cohort involved was small, these results corroborate our findings. In our study, a high PLR was associated with increased mortality. Note that we also observed a higher proportion of surgical therapeutic abstention in the group with high PLR value. In addition, patients with high PLR value were significantly older. This is in accordance with another study which demonstrated that the PLR increases with age [26]. Whether PLR identifies patients at high-risk of complications or patients who are contra-indicated for surgical procedure remains to be determined.

Intriguingly, even if patients with a high NLR tended to have higher rates of mortality, the difference did not reach statistical significance in our study. The analysis of the statistical power revealed that further studies on larger cohorts would be required before drawing any definitive conclusion. During inflammation, neutrophil count increases and lymphocyte decreases, resulting in an increase of the NLR [17]. Several studies have addressed the utility of the NLR in patients with acute mesenteric ischemia. The NLR was higher in patients with acute mesenteric ischemia or with non-vascular bowel necrosis compared to control patients with non-specific abdominal pain [17]. However, no difference was observed between patients with acute mesenteric ischemia and those with non-vascular bowel necrosis. Another study corroborated the potential diagnostic interest of the NLR, with significantly higher NLR in patients with acute mesenteric ischemia compared to healthy control individuals [19]. NLR value higher than 4.5 at admission yielded an area under the curve value of 0.790 (95% confidence interval 0.681–0.799, sensitivity 77%, specificity 72%). In addition, the NLR could help to identify differential diagnosis, as suggested by a significantly higher NLR value in patients with acute mesenteric ischemia compared with patients with acute appendicitis [18]. At last, the NLR was found to be an independent prognostic factor of patients with acute mesenteric arterial embolism and thrombosis (OR = 6.835, 95% confidence interval: 2.282–20.469, P = 0.001) [24].

The NLR and the PLR are systemic inflammatory markers which can be affected by the patients’ comorbidities [26–29]. We investigated the potential impact of the presence of diabetes, smoking, arterial hypertension, history of chronic inflammatory diseases or cancers and did not identify any significant difference among the subgroups.

Based on our results, several perspectives can be suggested. First, this was a retrospective single-center study and the size of the cohort may have limited the statistical power of the analysis. The number of patients per subgroups was sufficient to detect a difference on the 30-day mortality for the PLR. However, analysis of the statistical power for the NLR revealed that a higher number of patients per subgroups is required to confirm the results. Second, we investigated the 30-day outcomes and it would be of interest to extend this work on larger cohorts and longer follow-up periods. At last, the mechanistic relationship between high in-hospital PLR and 30-day mortality remain to be investigated.

## Conclusion

The PLR value at the in-hospital admission is a reliable and simple predictive factor of 30-day mortality in patients with acute mesenteric ischemia. Although further studies are required to establish a causal link between the PLR and outcomes of patients, it could be useful as a cheap and non-invasive prognostic biomarker in acute mesenteric ischemia.

## Supporting information

S1 Table Management of the acute mesenteric ischemia according to the PLR value. Values are expressed as n (%). PLR: platelet to lymphocyte ratio
(PDF)

S2 Table Management of the acute mesenteric ischemia according to the NLR value. Values are expressed as n (%). NLR: neutrophil to lymphocyte ratio
(PDF)

S3 Table Comparison of initial clinical characteristics according to the PLR value. Values are expressed as n (%). PLR: platelet to lymphocyte ratio
(PDF)

S4 Table Comparison of initial clinical characteristics according to the NLR value. Values are expressed as n (%). PLR: platelet to lymphocyte ratio
(PDF)

## References

[1] Karabulut K, Gul M, Dundar ZD, Cander B, Kurban S, Toy H. Diagnostic and prognostic value of procalcitonin and phosphorus in acute mesenteric ischemia. Ulus Travma Acil Cerrahi Derg. 2011;17(3):193–8. 21935794 10.5505/tjtes.2011.70493

[2] Alhan E, Usta A, Cekic A, Saglam K, Turkyilmaz S, Cinel A. A study on 107 patients with acute mesenteric ischemia over 30 years. Int J Surg. 2012;10(9):510–3. 10.1016/j.ijsu.2012.07.011 22885139

[3] Kassahun WT, Schulz T, Richter O, Hauss J. Unchanged high mortality rates from acute occlusive intestinal ischemia: six year review. Langenbecks Arch Surg. 2008;393(2):163–71. 10.1007/s00423-007-0263-5 18172675

[4] Block T, Nilsson TK, Bjorck M, Acosta S. Diagnostic accuracy of plasma biomarkers for intestinal ischaemia. Scand J Clin Lab Invest. 2008;68(3):242–8. 10.1080/00365510701646264 17934974

[5] Acosta S, Nilsson TK, Bjorck M. D-dimer testing in patients with suspected acute thromboembolic occlusion of the superior mesenteric artery. Br J Surg. 2004;91(8):991–4. 10.1002/bjs.4645 15286959

[6] Treskes N, Persoon AM, van Zanten ARH. Diagnostic accuracy of novel serological biomarkers to detect acute mesenteric ischemia: a systematic review and meta-analysis. Intern Emerg Med. 2017;12(6):821–36. 10.1007/s11739-017-1668-y 28478489 PMC5559578

[7] Derikx JP, Schellekens DH, Acosta S. Serological markers for human intestinal ischemia: A systematic review. Best Pract Res Clin Gastroenterol. 2017;31(1):69–74. 10.1016/j.bpg.2017.01.004 28395790

[8] Nunez J, Nunez E, Bodi V, Sanchis J, Minana G, Mainar L, et al Usefulness of the neutrophil to lymphocyte ratio in predicting long-term mortality in ST segment elevation myocardial infarction. Am J Cardiol. 2008;101(6):747–52. 10.1016/j.amjcard.2007.11.004 18328833

[9] Chan C, Puckridge P, Ullah S, Delaney C, Spark JI. Neutrophil-lymphocyte ratio as a prognostic marker of outcome in infrapopliteal percutaneous interventions for critical limb ischemia. J Vasc Surg. 2014;60(3):661–8. 10.1016/j.jvs.2014.03.277 24816510

[10] Tamhane UU, Aneja S, Montgomery D, Rogers EK, Eagle KA, Gurm HS. Association between admission neutrophil to lymphocyte ratio and outcomes in patients with acute coronary syndrome. Am J Cardiol. 2008;102(6):653–7. 10.1016/j.amjcard.2008.05.006 18773982

[11] Gibson PH, Croal BL, Cuthbertson BH, Small GR, Ifezulike AI, Gibson G, et al Preoperative neutrophil-lymphocyte ratio and outcome from coronary artery bypass grafting. Am Heart J. 2007;154(5):995–1002. 10.1016/j.ahj.2007.06.043 17967611

[12] Lareyre F, Raffort J, Le D, Chan HL, Houerou TL, Cochennec F, et al High Neutrophil to Lymphocyte Ratio Is Associated With Symptomatic and Ruptured Thoracic Aortic Aneurysm. Angiology. 2018;69(8):686–91. 10.1177/0003319717751758 29334754

[13] Massiot N, Lareyre F, Voury-Pons A, Pelletier Y, Chikande J, Carboni J, et al High Neutrophil to Lymphocyte Ratio and Platelet to Lymphocyte Ratio are Associated with Symptomatic Internal Carotid Artery Stenosis. J Stroke Cerebrovasc Dis. 2019;28(1):76–83. 10.1016/j.jstrokecerebrovasdis.2018.09.001 30268367

[14] Akboga MK, Canpolat U, Yayla C, Ozcan F, Ozeke O, Topaloglu S, et al Association of Platelet to Lymphocyte Ratio With Inflammation and Severity of Coronary Atherosclerosis in Patients With Stable Coronary Artery Disease. Angiology. 2016;67(1):89–95. 10.1177/0003319715583186 25922197

[15] Uzun F, Erturk M, Cakmak HA, Kalkan AK, Akturk IF, Yalcin AA, et al Usefulness of the platelet-to-lymphocyte ratio in predicting long-term cardiovascular mortality in patients with peripheral arterial occlusive disease. Postepy Kardiol Interwencyjnej. 2017;13(1):32–8. 10.5114/aic.2017.66184 28344615 PMC5364280

[16] Lareyre F, Carboni J, Chikande J, Massiot N, Voury-Pons A, Umbdenstock E, et al Association of Platelet to Lymphocyte Ratio and Risk of 30-Day Postoperative Complications in Patients Undergoing Abdominal Aortic Surgical Repair. Vasc Endovascular Surg. 2019;53(1):5–11. 10.1177/1538574418789046 30021492

[17] Tanrikulu Y, Sen Tanrikulu C, Sabuncuoglu MZ, Temiz A, Kokturk F, Yalcin B. Diagnostic utility of the neutrophil-lymphocyte ratio in patients with acute mesenteric ischemia: A retrospective cohort study. Ulus Travma Acil Cerrahi Derg. 2016;22(4):344–9. 10.5505/tjtes.2015.28235 27598606

[18] Aktimur R, Cetinkunar S, Yildirim K, Aktimur SH, Ugurlucan M, Ozlem N. Neutrophil-to-lymphocyte ratio as a diagnostic biomarker for the diagnosis of acute mesenteric ischemia. Eur J Trauma Emerg Surg. 2016;42(3):363–8. 10.1007/s00068-015-0546-4 26059561

[19] Toptas M, Akkoc I, Savas Y, Uzman S, Toptas Y, Can MM. Novel hematologic inflammatory parameters to predict acute mesenteric ischemia. Blood Coagul Fibrinolysis. 2016;27(2):127–30. 10.1097/MBC.0000000000000372 26258672

[20] Bala M, Kashuk J, Moore EE, Kluger Y, Biffl W, Gomes CA, et al Acute mesenteric ischemia: guidelines of the World Society of Emergency Surgery. World J Emerg Surg. 2017;12:38 10.1186/s13017-017-0150-5 28794797 PMC5545843

[21] Bradbury AW, Brittenden J, McBride K, Ruckley CV. Mesenteric ischaemia: a multidisciplinary approach. Br J Surg. 1995;82(11):1446–59. 10.1002/bjs.1800821105 8535792

[22] Tsokos GC, Liossis SN. Lymphocytes, cytokines, inflammation, and immune trafficking. Curr Opin Rheumatol. 1998;10(5):417–25. 9746856 10.1097/00002281-199809000-00004

[23] Altintas O, Altintas MO, Tasal A, Kucukdagli OT, Asil T. The relationship of platelet-to-lymphocyte ratio with clinical outcome and final infarct core in acute ischemic stroke patients who have undergone endovascular therapy. Neurol Res. 2016;38(9):759–65. 10.1080/01616412.2016.1215030 27477691

[24] Wang S, Liu H, Wang Q, Cheng Z, Sun S, Zhang Y, et al Neutrophil-to-Lymphocyte Ratio and Platelet-to-Lymphocyte Ratio Are Effective Predictors of Prognosis in Patients with Acute Mesenteric Arterial Embolism and Thrombosis. Ann Vasc Surg. 2018;49:115–22. 10.1016/j.avsg.2018.01.059 29428537

[25] Yilmaz EM, Carti EB. Prognostic factors in acute mesenteric ischemia and evaluation with Mannheim Peritonitis Index and platelet-to-lymphocyte ratio. Ulus Travma Acil Cerrahi Derg. 2017;23(4):301–5. 10.5505/tjtes.2016.00701 28762450

[26] Lin BD, Hottenga JJ, Abdellaoui A, Dolan CV, de Geus EJC, Kluft C, et al Causes of variation in the neutrophil-lymphocyte and platelet-lymphocyte ratios: a twin-family study. Biomark Med. 2016;10(10):1061–72. 10.2217/bmm-2016-0147 27690543 PMC5220440

[27] Hussain M, Babar MZM, Akhtar L, Hussain MS. Neutrophil lymphocyte ratio (NLR): A well assessment tool of glycemic control in type 2 diabetic patients. Pak J Med Sci. 2017;33(6):1366–70. 10.12669/pjms.336.12900 29492060 PMC5768826

[28] Gao Y, Wang WJ, Zhi Q, Shen M, Jiang M, Bian X, et al Neutrophil/lymphocyte ratio is a more sensitive systemic inflammatory response biomarker than platelet/lymphocyte ratio in the prognosis evaluation of unresectable pancreatic cancer. Oncotarget. 2017;8(51):88835–44. 10.18632/oncotarget.21340 29179480 PMC5687650

[29] Qin B, Ma N, Tang Q, Wei T, Yang M, Fu H, et al Neutrophil to lymphocyte ratio (NLR) and platelet to lymphocyte ratio (PLR) were useful markers in assessment of inflammatory response and disease activity in SLE patients. Mod Rheumatol. 2016;26(3):372–6. 10.3109/14397595.2015.1091136 26403379

