# Supplementary material for: Platelet to lymphocyte ratio as a predictive factor of 30-day mortality in patients with acute mesenteric ischemia
Source: PLoS One. 2019 Jul 17;14(7):e0219763. doi: 10.1371/journal.pone.0219763 (PMC6636734; doi:10.1371/journal.pone.0219763)
Supplement: S1 Table — Values are expressed as n (%). PLR: platelet to lymphocyte ratio (PDF) [file pone.0219763.s001.pdf]

Procedural characteristics	PLR<167.9 (n=26)	167.9<PLR<268.1 (n=27)	268.1<PLR<429.3 (n=27)	PLR>429.3 (n=26)	P value
**Localization of the vascular thrombosis**					
. Celiac trunk	3 (11.5%)	3 (11.1%)	2 (7.4%)	2 (7.7%)	0.93
. Mesenteric superior artery	18 (69.2%)	21 (77.8%)	17 (63%)	21 (80.8%)	0.45
. Mesenteric inferior artery	5 (19.2%)	1 (3.7%)	5 (18.5%)	2 (7.7%)	0.22
. Mesenteric vein	2 (7.7%)	1 (3.7%)	1 (3.7%)	0 (0%)	0.55
. Undetermined	1 (3.8%)	3 (11.1%)	6 (22.2%)	3 (11.5%)	0.24
**Surgical treatment**					
Intestinal resection					
. Jejunum resection	2 (7.7%)	1 (3.7%)	3 (11.1%)	1 (3.8%)	0.65
. Ileum resection	7 (26.9%)	6 (22.2%)	6 (22.2%)	7 (26.9%)	0.96
. Colon resection	8 (30.8%)	4 (14.8%)	8 (29.6%)	7 (26.9%)	0.52
Revascularization					
. Vascular bypass	1 (3.8%)	2 (7.4%)	2 (7.4%)	1 (3.8%)	0.89
. Endovascular repair	3 (11.5%)	0 (0%)	2 (7.4%)	0 (0%)	0.12
. Thrombectomy	5 (19.2%)	3 (11.1%)	3 (11.1%)	1 (3.8%)	0.38
Surgical therapeutic abstention	6 (23.1%)	14 (51.9%)	15 (55.6%)	15 (57.7%)	0.04

S1 table: Management of the acute mesenteric ischemia according to the PLR value.

Values are expressed as n (%).

PLR: platelet to lymphocyte ratio
