# Supplementary material for: Platelet to lymphocyte ratio as a predictive factor of 30-day mortality in patients with acute mesenteric ischemia
Source: PLoS One. 2019 Jul 17;14(7):e0219763. doi: 10.1371/journal.pone.0219763 (PMC6636734; doi:10.1371/journal.pone.0219763)
Supplement: S2 Table — Values are expressed as n (%). NLR: neutrophil to lymphocyte ratio (PDF) [file pone.0219763.s002.pdf]

| Procedural characteristics | NLR<7.5 (n=26) | 7.5<NLR<14.2 (n=27) | 14.2<NLR<21.2 (n=27) | NLR>21.2 (n=26) | P value |
|---|---|---|---|---|---|
| **Localization of the vascular thrombosis** | | | | | |
| . Celiac trunk | 1 (3.8%) | 4 (14.8%) | 1 (3.7%) | 4 (15.4%) | 0.27 |
| . Mesenteric superior artery | 19 (73.1%) | 18 (66.7%) | 21 (77.8%) | 19 (73.1%) | 0.84 |
| . Mesenteric inferior artery | 2 (7.7%) | 5 (18.5%) | 4 (14.8%) | 2 (7.7%) | 0.54 |
| . Mesenteric vein | 2 (7.7%) | 1 (3.7%) | 1 (3.7%) | 0 (0%) | 0.55 |
| . Undetermined | 2 (7.7%) | 3 (11.1%) | 3 (11.1%) | 5 (19.2%) | 0.63 |
| **Surgical treatment** | | | | | |
| Intestinal resection | | | | | |
| . Jejunum resection | 0 (0%) | 3 (11.1%) | 2 (7.4%) | 2 (7.7%) | 0.42 |
| . Ileum resection | 4 (15.4%) | 7 (25.9%) | 7 (25.9%) | 8 (30.8%) | 0.62 |
| . Colon resection | 6 (23.1%) | 6 (22.2%) | 7 (25.9%) | 8 (30.8%) | 0.89 |
| Revascularization | | | | | |
| . Vascular bypass | 1 (3.8%) | 2 (7.4%) | 2 (7.4%) | 1 (3.8%) | 0.89 |
| . Endovascular repair | 2 (7.7%) | 2 (7.4%) | 0 (0%) | 1 (3.8%) | 0.51 |
| . Thrombectomy | 4 (15.4%) | 5 (18.5%) | 2 (7.4%) | 1 (3.8%) | 0.30 |
| Surgical therapeutic abstention | 9 (34.6%) | 11 (40.7%) | 15 (55.6%) | 15 (57.7%) | 0.36 |

S2 table: Management of the acute mesenteric ischemia according to the NLR value.

Values are expressed as n (%).

NLR: neutrophil to lymphocyte ratio
