# Supplementary material for: Platelet to lymphocyte ratio as a predictive factor of 30-day mortality in patients with acute mesenteric ischemia
Source: PLoS One. 2019 Jul 17;14(7):e0219763. doi: 10.1371/journal.pone.0219763 (PMC6636734; doi:10.1371/journal.pone.0219763)
Supplement: S3 Table — Values are expressed as n (%). PLR: platelet to lymphocyte ratio (PDF) [file pone.0219763.s003.pdf]

Clinical characteristics	PLR<167.9<br><br>(n=26)	167.9<PLR<268.1<br><br>(n=27)	268.1<PLR<429.3<br><br>(n=27)	PLR>429.3<br><br>(n=26)	P value
Age	67 (59 – 83)	78 (50 – 85)	83 (66 - 89)	84 (71 – 90)	0.03
Diabetes	6 (22.2%)	5 (18.5%)	6 (22.2%)	6 (23.1%)	0.22
Arterial hypertension	15 (55.6%)	16 (59.3%)	17 (63%)	15 (57.7%)	0.97
Smoking	9 (33.3%)	5 (18.5%)	11 (40.7%)	8 (30.8%)	0.35
History of inflammatory disease	1 (3.7%)	3 (11.1%)	2 (7.4%)	1 (3.8%)	0.67
History of cancer	2 (7.4%)	4 (14.8%)	6 (22.2%)	2 (7.7%)	0.34

S3 Table: Comparison of initial clinical characteristics according to the PLR value.

Values are expressed as n (%).

PLR: platelet to lymphocyte ratio
