# Supplementary material for: Platelet to lymphocyte ratio as a predictive factor of 30-day mortality in patients with acute mesenteric ischemia
Source: PLoS One. 2019 Jul 17;14(7):e0219763. doi: 10.1371/journal.pone.0219763 (PMC6636734; doi:10.1371/journal.pone.0219763)
Supplement: S4 Table — Values are expressed as n (%). PLR: platelet to lymphocyte ratio (PDF) [file pone.0219763.s004.pdf]

**Clinical characteristics**	**PLR<167.9 (n=26)**	**167.9<PLR<268.1 (n=27)**	**268.1<PLR<429.3 (n=27)**	**PLR>429.3 (n=26)**	**P value**
Age	79 (60 – 85)	74 (61 – 88)	81 (60 – 90)	81 (66 – 89)	0.53
Diabetes	6 (23.1%)	3 (11.1%)	6 (22.2%)	8 (30.8%)	0.38
Arterial hypertension	16 (61.5%)	15 (55.6%)	20 (74.1%)	12 (46.2%)	0.17
Smoking	3 (11.5%)	2 (7.4%)	5 (18.5%)	5 (19.2%)	0.60
History of inflammatory disease	0 (0%)	1 (3.7%)	3 (11.1%)	3 (11.5%)	0.25
History of cancer	1 (3.8%)	5 (18.5%)	5 (18.5%)	3 (11.5%)	0.32

S4 Table: Comparison of initial clinical characteristics according to the NLR value.

Values are expressed as n (%).

PLR: platelet to lymphocyte ratio
